# DNA methylation within the I.4 promoter region correlates with CYPl19A1 gene expression in human *ex vivo* mature omental and subcutaneous adipocytes

**DOI:** 10.1186/1471-2350-14-87

**Published:** 2013-08-30

**Authors:** Joshua R Lewis, Tegan J McNab, Lawrence J Liew, Jeremy Tan, Phillip Hudson, Jenny Z Wang, Richard L Prince

**Affiliations:** 1 School of Medicine and Pharmacology, University of Western Australia, Nedlands, WA, Australia; 2 Department of Endocrinology and Diabetes, Sir Charles Gairdner Hospital, Nedlands, WA, Australia; 3 Department of General Surgery, Sir Charles Gairdner Hospital, Nedlands, WA, Australia; 4 QIAGEN Pty. Ltd, Doncaster, VIC, Australia

**Keywords:** Aromatase gene expression, DNA methylation, Circulating estradiol, Bone structure, Epigenetics

## Abstract

**Background:**

DNA methylation at specific CpG sites within gene promoter regions is known to regulate transcriptional activity *in vitro*. In human adipose tissue, basal transcription of the aromatase (CYP19A1) gene is driven primarily by the I.4 promoter however the role of DNA methylation in regulating expression in *ex vivo* mature adipocytes is unknown. This observational study reports the correlation of DNA methylation within the I.4 promoter region of human mature subcutaneous and omental adipocytes with aromatase expression and body composition measures.

**Methods:**

Omental and subcutaneous adipose tissue were collected from 25 obese subjects undergoing bariatric surgery and the mature adipocyte fraction purified. DNA methylation status of 5 CpG sites within a 550 base pair region encompassing the transcription start site (TSS) of promoter I.4 was determined using pyrosequencing. Relative aromatase and I.4 promoter specific mRNA expression was determined by qRT-PCR and whole body DXA performed in 25 participants.

**Results:**

Site-specific DNA methylation varied from 21 ± 10% to 81 ± 11%. In omental adipocytes percentage methylation at the I.4.1 and I.4.2 CpG sites, but not other nearby sites, was negatively correlated with relative aromatase mRNA expression (R = - 0.52, P = 0.017 and R = - 0.52, P = 0.015). In contrast subcutaneous adipocytes percentage DNA methylation at the I.4.3 and I.4.5 sites were positively correlated with relative aromatase mRNA expression (R = 0.47, P = 0.022 and R = 0.55, P = 0.004). In a small subset of patients DNA methylation at the I.4.5 site was also positively correlated with whole body lean mass, bone mineral content and density.

**Conclusions:**

In conclusion in mature adipocytes, the primary source of estradiol after menopause, increasing DNA methylation was correlated with aromatase mRNA expression and thus estradiol biosynthesis. These findings support a tissue-specific epigenetic regulation of the basal promoter activity in mature adipocytes; the mechanisms influencing this regulation and its physiological role remain to be elucidated.

## Background

Estrogen biosynthesis is catalysed by the aromatase enzyme which converts androstendione to estrone and testosterone to estradiol [1,2]. Aromatase is a cytochrome P450 enzyme produced by the human *CYP19A1* gene located on chromosome 15q21.2 [3]. The gene comprises of an upstream regulatory region spanning approximately 93 kb and a downstream coding region consisting of 9 coding exons, II-X, spanning 30 kb [4]. Aromatase expression has been detected in the ovary, placenta, breast, testes, adipose tissue, bone, peripheral blood leucocytes and brain [4,5]. However adipose tissue is the primary source of estrogen in women after menopause and is important in maintaining bone remodelling and regulating adipose mass. Tissue specific aromatase gene expression is determined in part by tissue-specific promoters in the regulatory regions upstream of the untranslated first exon which gives rise to transcripts with unique 5′ non-coding termini [6-8].

In human adipose tissue, basal transcription of aromatase has been demonstrated to be driven primarily through the I.4 promoter [7,9-11] with a 500 bp region of the aromatase I.4 promoter located 330 bp upstream to 170 bp downstream of the transcription start site (TSS) been shown to regulate glucocorticoid responsiveness [9]. DNA methylation is an epigenetic mark which involves the addition of a methyl group to the fifth carbon of the cytosine ring, forming 5-methylcytosine. The reaction is catalysed by DNA methyltransferases and primarily occurs in a 5′ CpG dinucleotide context [12]. Typically DNA methylation is associated with repressing gene expression [13] and activation of genes has been attributed to the demethylation of critical CpG loci both *in vitro* and *in vivo*[12,14-17] however intragenic DNA methylation paradoxically has been shown to be associated with increased gene expression [18-20]. DNA methylation has also been suggested to regulate placental [21] and ovarian [22] aromatase expression in cattle, while a recent *in vitro* study [11] suggested an inverse association between DNA methylation in the aromatase I.4 promoter region and aromatase gene expression in human breast adipose fibroblasts and breast cell lines however the relationship between DNA methylation and other cell types has not been investigated.

In men and after menopause in women pre-adipocytes and mature adipocytes are considered the primary source of circulating levels of estradiol after menopause [23]. Endogenous production of estradiol and therefore its regulation in adipose tissue has important clinical implications for a range of diseases including breast cancer [24], Alzheimer’s disease [25] and fracture [26]. Previous studies by our group and others have investigated the association of genetic polymorphisms within the aromatase gene with estradiol levels, bone mineral density, and fracture risk [26-31], however little is known regarding the role of epigenetic regulation of the aromatase gene.

We therefore used human *ex vivo* mature adipocytes from the subcutaneous and omental sites to test the association of DNA methylation within the I.4 promoter and gene expression.

## Methods

### Subjects

Patients undergoing bariatric surgery at Sir Charles Gairdner Hospital were invited to participate in the study. Twenty five participants were recruited for the study and adipose tissue samples collected at surgery. Each participant gave written informed consent and the study was approved by the institutional ethics committee of Sir Charles Gairdner Hospital.

### Body composition and bone mineral density

Whole body dual x-ray absorptiometry (DXA) was performed in 10 participants during a clinic visit post-surgery and headless bone area, mineral content and density as well as whole body bone free lean mass and fat mass on a fan-beam densitometer (Hologic Acclaim 4500A; Hologic Corp, Waltham, Mass) were measured.

### Adipocyte isolation

Omental and subcutaneous adipose tissue was taken during bariatric surgery. Adipose tissue was rapidly processed after surgery using a modified protocol [32]. Briefly, connective tissue, blood vessels and fibrous materials were removed from the adipose tissue samples. The tissue were then homogenised and incubated in collagenase digest solution (25 mM HEPES, 5 mM glucose, 120 mM NaCl, 50 mM KCl, 7.5 mM CaCl_2_, 3 mg/ml Type II Collagenase, Sigma) with a ratio of 4:1 at 37°C in a 180 rpm shaker for 1.5 hours. The digest was then passed through a 500 μm nylon mesh and spun at 700 g for 10 min. After gentle agitation of the culture for 30 seconds the floating layer of cells that resurfaced (mature adipocytes) were then collected and the procedure repeated twice more. This method of isolating adipocytes has been shown to result in ~3-4 contaminating pre-adipocytes per 1,000 mature adipocytes, as described by others [33]. Cells were stored at −80°C in RNAlater prior to extraction. RNA samples were extracted using the AllPrep DNA/RNA and protein kit (Qiagen, VIC, Australia) according to the manufacturer’s instructions. RNA concentrations were then determined by spectrophotometry (ND-1000, Nanodrop Technologies, USA) with a 260/280 of 1.8 or greater. Where available mature adipocytes from 36 of the original 50 samples were re-extracted using the AllPrep Mini Kit (QIAGEN) for PI.3 and PI.4 transcript expression analysis.

### Pyrosequencing primer design

**Figure 1:**
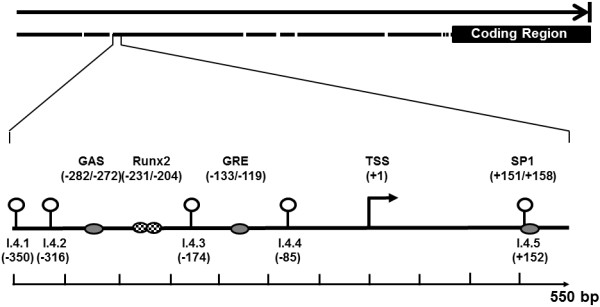
**Location of the 5 CpG sites analysed within the I.4 promoter region of the aromatase gene.** Numbers indicate positions of CpG sites analysed relative to the transcription start site (TSS, +1) of I.4, sites 1- 5n refer to I.4.1 – 5 respectively. *Cis*-acting elements are shown in circles (5, 14, 37). GAS, interferon γ activation site; GRE, glucocorticoid response element; Runx2, Runt-related transcription factor 2.

Pyrosequencing assays were designed using the PyroMark assay design software (version 2.0, QIAGEN). The most recent version of the genomic DNA sequence was imported into the assay design software (accession number L21982). The region of interest for this study was identified as being 350 bp upstream of the transcription start site to 200 bp downstream as this region has previously been identified to be responsible for glucocorticoid stimulated aromatase gene expression in primary hepatocytes and adipose derived stromal cells [9]. This region encompasses a glucocorticoid response element from −133/119 bp and an SP1 binding site +151/158 responsible for glucocorticoid responsiveness [9] as well as a RUNX2 binding site from −231/204 bp [34] and a interferon-gamma activation site element from −282/272 bp [35] which are involved in activation of I.4 promoter specific transcription (Figure 1). Primers were designed for the 5 CpG sites within this region. *In silico* bisulfite conversion was performed by converting all CG’s in the gDNA sequence to YG’s and any remaining C’s to T’s. Sites I.4.1 (−350 bp), I.4.2 (−316 bp), I.4.3 (−174 bp), I.4.4 (−85 bp) and I.4.5 (+152 bp) correspond to CpG sites 4, 5, 6, 7 and 8 respectively, described by Knower et al. [11]. The primer sets which resulted in the highest primer score based on the predictive likelihood of primers forming duplexes and hairpins, mispriming or the amplicon looping for sites 1, 2 and 5 were selected (Table 1).

**Table 1:** Primer sequences for primers used in the PCR and pyrosequencing reactions

	**Primer name**	**Sequence (5′ to 3′)**
I.4.1, I.4.2^a^	I.4a PCR F	GGAATGGTGAGAGTTTGGTTAATG
	I.4a PCR R	*CAAAAAACACCCTAAAATATAACCTACAA
	I.4a Seq.^b^	GTAAAGTGTTTGTTTTTTTATAGT
I.4.3, I.4.4^a^	I.4c PCR F	GGAATGGTGAGAGTTTGGTTAATG
	I.4c PCR R	*CAAAAAACACCCTAAAATATAACCTACAA
	I.4c Seq.^b^	CTACAATAAAAACACATTTCTT
I.4.5	I.4b PCR F	*AGGAGATTTTTGATTTATGTGGGGTTATG
	I.4b PCR R	ACTCAAACTCCAAAAACTTACCTAAT
	I.4b Seq.^b^	CCCATCACATCACTC

^a^Encompasses two CpG sites upstream of the promoter. ^b^Sequencing primer. *Biotinylated at 5′ end and HPLC purified to enable capture of the PCR products by the PyroMark vacuum station filter probes.

### Pyrosequencing assay

Unmethylated cytosines were converted to uracil using the EpiTect bisulfite kit (QIAGEN) according to the manufacturer’s instructions. Bisulfite converted DNA was determined by spectrophotometry (ND-1000, Nanodrop Technologies, USA) and 40 ng was amplified using the PyroMark PCR kit (QIAGEN) as per the manufacturer’s instructions. Cycling conditions were as follows: 95°C for 15 min, 45 cycles of 94°C for 30 s, 56°C for 30 s and 72°C for 30 s, followed by a final extension step at 72°C for 10 mins. 2 μl of well-mixed streptavidin sepharose high performance slurry (GE Healthcare) and 40 μl of binding buffer (QIAGEN) was diluted in 18 μl of water in each well, followed by addition of 20 μl of PCR product generated previously. The mixture was vortexed for 5 mins at 1,400 rpm and the 24-well plate containing the PCR product-streptavidin mix was placed on the PyroMark Vacuum station. With the vacuum on, the filter probes were placed in sterile water for 10 s and inserted into the 24-well plate containing the PCR product-streptavidin mix to capture the double stranded PCR (dsPCR) product. The dsPCR product was washed in 70% ethanol solution for 5 s and then denaturation solution (QIAGEN) for 5 s. The single stranded biotinylated template was washed in 1 × wash buffer (QIAGEN) for 10 s and released by agitation. The template DNA was annealed to the sequencing primer by incubating the PyroMark Q24 plate at 80°C for 2 min. The PyroMark Q24 cartridge was loaded with PyroGold Q24 reagents, according to pre-run information (PyroMark Q24 software). Each run contained at least 1 internal bisulfite conversion control whereby C bases not followed by a G (typically not methylated) should be fully converted to T after bisulfite treatment. All runs also included a no template control. The proportion of DNA molecules methylated at each CpG site was automatically calculated by the PyroMark Q24 software and expressed as a percentage. As previously reported, the quantitative reproducibility of technical replicates amplified by the same PCR is approximately 2% and variation induced by different bisulfite treatments and/or separate PCR amplifications is approximately 5% [36].

### Relative aromatase mRNA expression

mRNA from the adipocytes was reverse transcribed using the QuantiTect reverse transcriptase kit (QIAGEN), according to manufacturer’s instructions, including a genomic DNA elimination step and adding the maximum amount of template mRNA possible. Total aromatase mRNA expression was then determined using the QuantiFast SYBR green PCR kit (QIAGEN) and QuantiTect primer assay (Hs_CYP19A1_2_SG; QIAGEN) according to the manufacturer’s instructions, in an iQ5 real time PCR thermal cycler (BioRad). Briefly, each reaction consisted of 1× QuantiFast Master mix, 1× QuantiTect primer assay, 4 μl of cDNA previously diluted 1:2 with RNase-free H_2_O and made up to 15 μl with RNase-free H_2_O. The primers amplified an 82 bp region of aromatase (accession numbers: NM_000103, NM_031226). Aromatase mRNA expression was normalised to 18S rRNA gene expression, which was determined using the same conditions as above but with a different QuantiTect Primer Assay (Hs_RRNA18S_1_SG; QIAGEN). Where available (n = 36) excess adipocytes were stored in RNAlater® (QIAGEN) were re-extracted and relative promoter expression was calculated using RT-PCR using I.4 and I.3 promoter specific forward primers with a common reverse primers as described by Demura et al. [37].

### Statistical analysis

Differences between groups were investigated using independent samples T-Tests and tissue depots by paired sample T-Tests. Spearman’s Rank correlation coefficient (R) was used to investigate the relationships between percentage DNA methylation and relative aromatase mRNA expression, age, body mass index, lean mass, fat mass and bone phenotypes. All correlations observed were tested with and without outliers and with and without men to account for potential gender differences. P values < 0.05 were considered statistically significant. Results were analysed using SPSS (PASW Statistics, version 18). Results are given as mean ± standard deviation.

## Results

### Cohort characteristics

The subjects consisted of 22 women and 3 men undergoing bariatric surgery. The mean age was 46.1 ± 13.8 years, height 166.7 ± 8.8 cm, weight 121.3 ± 24.2 kg and body mass index (BMI) 43.4 ± 8.1 kg/m^2^.

### DNA methylation of CpG sites within the regulatory region of the I.4 promoter

**Figure 2:**
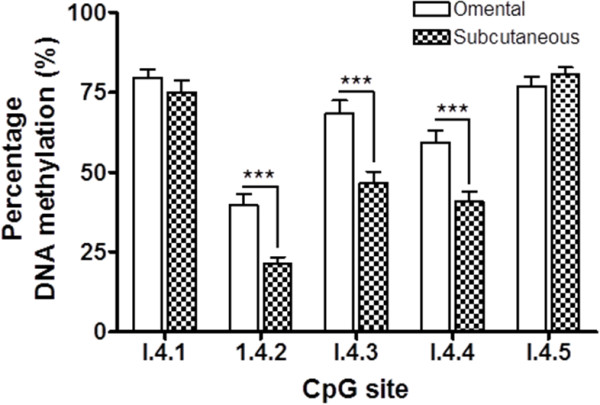
Mean ± SEM of percentage DNA methylation at the 5 CpG sites within the I.4 promoter of subcutaneous adipocytes compared to omental adipocytes. ^***^ represents significantly different from omental adipocytes P < 0.001.

The four CpG sites upstream of the TSS identified by the PyroMark assay design software were I.4.1 and I.4.2, I.4.3 and I.4.4 while the CpG site I.4.5 was located downstream of the TSS (Figure 1). The percentage DNA methylation of the mature adipocytes from the omental and subcutaneous depots for the 5 CpG sites studied is shown in Figure 2. Three of the upstream CpG sites; (I.4.2 – I.4.4) had significantly different percentage DNA methylation between omental and subcutaneous adipocytes.

### Site specific methylation and relative total mRNA expression

#### Omental adipocytes

Total relative aromatase expression in omental adipocytes was 0.42 ± 0.15 in omental adipocytes. In omental adipocytes percentage DNA methylation at CpG sites I.4.1 and I.4.2 was negatively correlated with relative total aromatase mRNA expression (R = −0.516, P = 0.017 and R = −0.522, P = 0.015 respectively). DNA methylation was not significantly correlated with total aromatase expression at sites I.4.3 (R = −0.203, P = 0.391), I.4.4 (R = −0.131, P = 0.582) or I.4.5 (R = −0.263, P = 0.237). The association between DNA methylation and total aromatase expression became non-significant at the I.4.1 site after the exclusion of men from the analysis (R = −0.462, P = 0.054). Similarly the correlation between the I.4 promoter specific mRNA expression and 1.4.1 and 1.4.2 DNA methylation failed to reach significance (R = −0.357, p = 0.199 and −0.262, P = −0.311).

#### Subcutaneous adipocytes

**Figure 3:**
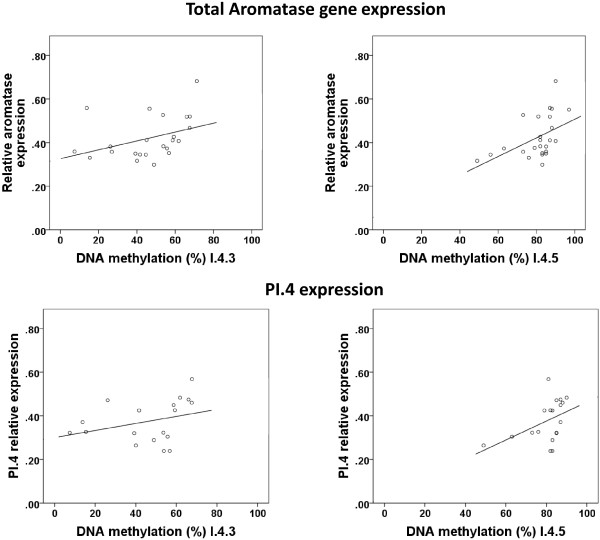
**Correlation between percentage DNA methylation at CpG sites 3 and 5 with total relative aromatase mRNA expression (n = 25) and I.4 promoter transcript aromatase expression (n = 19) in human subcutaneous adipocytes.** Top left: Correlation between methylation percentage at the I.4.3 CpG site and total aromatase expression in (R = 0.474, P = 0.022). Top right: Correlation between methylation percentage at the I.4.5 CpG site and total aromatase expression (R = 0.549, P = 0.004). Bottom left: Correlation between methylation percentage at the I.4.3 CpG site and PI.4 aromatase expression (R = 0.449, P = 0.062). Bottom right: Correlation between methylation percentage at the I.4.5 site and PI.4 aromatase expression (R = 0.476, P = 0.040).

Total relative aromatase expression in subcutaneous adipocytes was 0.42 ± 0.15in subcutaneous adipocytes. In subcutaneous adipocytes percentage DNA methylation at the I.4.3 and I.4.5 CpG sites were positively correlated with relative total aromatase mRNA expression (Figure 3). DNA methylation was not significantly correlated with relative total aromatase expression at sites I.4.1 (R = 0.302, P = 0.142), I.4.2 (R = 0.047, P = 0.824) or I.4.4 (R = −0.063, P = 0.774). Similarly a positive correlation was also seen between DNA methylation and the I.4 promoter specific relative gene expression (Figure 3). The correlation between the I.4.5 CpG site and PI.4 aromatase expression remained significant after the exclusion of men (R = 0.473, P = 0.047). There were no correlations between percentage DNA methylation of the I.4 promoter CpG sites and relative PI.3 transcript expression.

#### Site specific methylation, gene expression and phenotype

Methylation levels at the 5 CpG sites were not associated with age, weight or BMI in either tissue type. In subcutaneous adipocytes CpG methylation at the I.4.4 methylation was significantly lower in males than females (21% vs. 39%), there were no other differences between males and females at other sites. There was no difference in total or I.4 promoter specific relative gene expression in either depot. In 10 participants with whole body DXA (9 women and 1 man) subcutaneous adipocytes I.4.5 methylation percentage was correlated with increased headless whole body bone mineral content (R = 0.783, P = 0.007), bone mineral density (R = 0. 0.636, P = 0.048) and lean mass (R = 0.661, P = 0.038), but not whole body bone area, lean or fat mass (data not shown).

## Discussion

In this study the correlation between DNA methylation determined by pyrosequencing within the regulatory region of the I.4 promoter region and total aromatase gene expression is extended to specific CpG sites from *ex vivo* human subcutaneous and omental mature adipocytes. The use of these primary fully differentiated cells allows a “snapshot” of the actual tissue specific relationship between DNA methylation and gene expression. Specifically we identified a negative correlation between DNA methylation at 2 CpG sites (I.4.1 and I 4.2) upstream of the aromatase promoter with aromatase mRNA expression in omental adipocytes. While in subcutaneous adipocytes percentage DNA methylation at I.4.3 site upstream of the transcription start site (TSS) and the I.4.5 site downstream of the TSS within the transcribed region of the gene were positively correlated with total aromatase mRNA expression. This “snapshot” of the DNA methylation status and gene expression in mature adipocytes expressing aromatase extends upon the findings of others using *in vitro* studies and supports the hypothesis that DNA methylation may play a role in tissue specific estrogen production.

Many epigenetic studies of DNA methylation and gene expression focus on genes with large CpG islands spanning the promoter regions of transcriptional initiation sites [38] and negative correlations between DNA methylation of CpG islands in the region of promoters and gene expression have been well described. However “non-classical” mechanisms are poorly understood. Given that the promoter regions of the aromatase gene does not contain CpG islands and is relatively CpG poor in comparison to other genomic regions [21,39-41] it is unlikely that “classical” epigenetic regulation is occurring at this site. An example of epigenetic regulation of a CpG poor promoter is that of the cytochrome p450 27B1 (CYP27B1) gene promoter where 13 CpG sites around the transcription start site are either hypermethylated by vitamin D binding to an upstream regulatory site or demethylated in the presence of parathyroid hormone in MCT cells [42].

Two DNA methylation sites (I.4.1, I.4.2) that are 34 bp upstream from the glucocorticoid response element were negatively correlated with total aromatase gene expression in omental adipocytes. These findings are similar to those reported by Knower et al. [11].

Unlike omental adipocytes a strong positive correlation was observed between DNA methylation I.4.5 CpG sites and a somewhat weaker correlation with the I.4.3 site and aromatase mRNA expression in subcutaneous adipocytes. While DNA methylation within transcribed regions has previously been correlated with increased gene expression the mechanisms still remain uncertain [18-20]. A number of other potential models that may explain the correlation between DNA methylation within the transcribed gene regions and aromatase expression in mature adipocytes exist, including regulation of alternative promoter initiation [43], heterogeneity amongst the mature adipocyte population purified or splicing [44,45] which is particularly likely given the tissue specific promoter usage of the aromatase gene [6] and the proximity of the I.4.5 CpG site to the splice junction. DNA methylation at the I.4.5 CpG site in subcutaneous adipocytes was also significantly positively associated with bone and body composition phenotypes. However caution must be taken in interpreting these findings given the nature of the cohort and the small number of participants with whole body composition and bone mineral density.

The strongest correlation between DNA methylation similarly was observed at the intragenic I.4.5 site located within a SP1 binding site that has previously been demonstrated to be important regulatory site for glucocorticoid stimulated aromatase gene expression in primary fetal hepatocytes and adipose derived stromal cells [9]. SP1 itself is an important regulator of CpG methylation status and when bound is thought to lead to hypomethylation enhanced transcription [46]. Interestingly, DNA methylation within the SP1 binding site does not appear to block SP1 binding [47], however it may be possible that DNA methylation within the SP1 binding site may block transcriptional repressors such as R1(RAM2/CDCA7L/JPO2) or E2F-associated phosphoprotein (EAPP) that compete with SP1 to bind to the SP1 binding site and have been reported to repress glucocorticoid dependant activation in other genes [48] further supporting the findings of others that adipocytes from subcutaneous tissue have greater responsiveness to glucocorticoids than omental tissue-derived adipocytes [23]. However further studies are needed to confirm the mechanism influencing these findings and its physiological role in estrogen production.

The strengths of this study was the use of matched omental and subcutaneous adipocytes, a purified homogenous *ex vivo* cell type to determine the relationship of DNA methylation within the I.4 promoter region with aromatase expression from a large number of human *ex vivo* samples not exposed to environmental hypomethylating agents in cell culture or extended passages. This model is therefore likely to represent endogenous relationships in mature adipocytes which are the primary extra-glandular source of estrogen in both men and women. The study also used pyrosequencing which is considered the “gold standard” for determining DNA methylation, in particular the inbuilt quality controls for bisulfite treatment [49] gives highly reproducible results [50] thus enabling quantitation of DNA methylation percentage at several CpG sites within close proximity [36].

An obvious limitation of the study is its observational nature however as yet no method of altering site specific DNA methylation status exists in primary human cells and hypo-methylation agents such as 5-aza-dC do not appear to hypomethylate CpG sites within the I.4 region of the aromatase gene *in vitro*[11]. Therefore currently the technology is not available to determine whether site specific DNA methylation actually alters gene expression in these cells.

## Conclusions

These data demonstrate that DNA methylation of specific CpG sites across the I.4 promoter region is variable exhibiting both positive and negative correlations with aromatase expression in mature *ex vivo* adipocytes depending on the tissue depot. In addition DNA methylation at one CpG site within the transcribed region in subcutaneous adipocytes was correlated with body composition and bone mineral density as well as gene expression. These data further support the concept that epigenetic mechanisms may be involved in the regulation of aromatase expression and may be important in the determination of body composition by mechanisms yet to be identified.

## Competing interests

The authors have read the journal’s policy and have the following conflicts: JRL, TJM, LJL, JZW, JT and RLP have nothing to declare. PH is an employee of QIAGEN Pty Ltd.

## Authors’ contributions

Authors obtaining funding included RLP and JRL; Authors participated in conception and design were JRL, TJM, PH, LJL, JW and RLP; Authors carried out data analysis and interpretation included JRL, TJM, PH, JZW, LJL and RLP; Authors who did drafting of the manuscript and critical revision were JRL, TJM, PH, JT, JZW, LJL and RLP. All authors have read and approved the final manuscript.

## Pre-publication history

The pre-publication history for this paper can be accessed here:

http://www.biomedcentral.com/1471-2350/14/87/prepub
